# Preoperative Biliary Drainage in Cases of Borderline Resectable Pancreatic Cancer Treated with Neoadjuvant Chemotherapy and Surgery

**DOI:** 10.1155/2016/7968201

**Published:** 2016-01-06

**Authors:** Tomofumi Tsuboi, Tamito Sasaki, Masahiro Serikawa, Yasutaka Ishii, Teruo Mouri, Akinori Shimizu, Keisuke Kurihara, Yumiko Tatsukawa, Eisuke Miyaki, Ryota Kawamura, Ken Tsushima, Yoshiaki Murakami, Kenichiro Uemura, Kazuaki Chayama

**Affiliations:** ^1^Department of Gastroenterology and Metabolism, Applied Life Science, Institute of Biomedical & Health Science, Hiroshima University, 1-2-3 Kasumi, Minami-ku, Hiroshima 734-8551, Japan; ^2^Department of Surgery, Institute of Biomedical and Health Sciences, Hiroshima University, 1-2-3 Kasumi, Minami-ku, Hiroshima 734-8551, Japan

## Abstract

*Objective*. To elucidate the optimum preoperative biliary drainage method for patients with pancreatic cancer treated with neoadjuvant chemotherapy (NAC).* Material and Methods*. From January 2010 through December 2014, 20 patients with borderline resectable pancreatic cancer underwent preoperative biliary drainage and NAC with a plastic or metallic stent and received NAC at Hiroshima University Hospital. We retrospectively analyzed delayed NAC and complication rates due to biliary drainage, effect of stent type on perioperative factors, and hospitalization costs from diagnosis to surgery.* Results*. There were 11 cases of preoperative biliary drainage with plastic stents and nine metallic stents. The median age was 64.5 years; delayed NAC occurred in 9 cases with plastic stent and 1 case with metallic stent (*p* = 0.01). The complication rates due to biliary drainage were 0% (0/9) with metallic stents and 72.7% (8/11) with plastic stents (*p* = 0.01). Cumulative rates of complications determined with the Kaplan-Meier method on day 90 were 60% with plastic stents and 0% with metallic stents (log-rank test, *p* = 0.012). There were no significant differences between group in perioperative factors or hospitalization costs from diagnosis to surgery.* Conclusions*. Metallic stent implantation may be effective for preoperative biliary drainage for pancreatic cancer treated with NAC.

## 1. Introduction

Radical surgery is the treatment of choice for pancreatic cancer. However, since pancreatic cancer is already at an advanced stage at the time of diagnosis in many cases, surgery may not be possible, and it tends to have a poor prognosis [1]. Combined modality therapy that includes both surgical resection and either chemotherapy or radiation therapy is important for improving the long-term prognosis of pancreatic cancer. Large-scale randomized studies of postoperative adjuvant chemotherapy have confirmed its efficacy, and the outcomes of resected pancreatic cancer are improving [2–5]. However, there is still insufficient evidence to support preoperative combined modality therapy.

The metallic stent (MS) is the standard stent used in biliary drainage for bile duct obstruction due to pancreatic head cancer in cases of unresectable pancreatic cancer since it has a longer patency period and is associated with fewer complications than the plastic stent (PS) [6]. However, there is no standard view on the stent to use for preoperative biliary drainage (PBD) in cases of resectable pancreatic cancer. van der Gaag et al. reported that PBD was unnecessary in cases in which early surgery (within 1 week) was possible [7]. However, in cases in which early surgery is not possible, PBD is considered necessary because of the waiting period until surgery can be performed. A PS has generally been used for PBD in cases in which surgery is to be performed, given the effect of MS placement on surgery as well as on cost [8–11]. However, the recent increase in the rate of bile duct-related complications is due to the lengthening of the preoperative period because of neoadjuvant chemotherapy (NAC) and the fact that NAC is now performed in cases of borderline resectable (BR) pancreatic cancer [12–18].

Thus, the purpose of the present study was to elucidate the optimum drainage technique for PBD performed in cases of pancreatic cancer treated with NAC.

## 2. Materials and Methods

### 2.1. Subjects

The subjects were 20 patients with BR cancer who underwent NAC with PBD at Hiroshima University Hospital between January 2010 and December 2014. Based on the drainage procedures performed, patients were divided into a plastic stent (PS) group and a metallic stent (MS) group. We then investigated delayed NAC and the rate of complications due to the use of bile duct drainage (retrograde cholangitis, stent occlusion, and stent migration); the effect of stent type on perioperative factors (amount of blood loss, operative time, and postoperative hospitalization) and postoperative complications; and the cost of hospitalization from diagnosis to surgery (excluding outpatient chemotherapy). This study received approval from the Hiroshima University Hospital Ethics Committee.

### 2.2. Diagnostic Strategy and Preoperative Biliary Drainage

Prior to bile duct drainage, all subjects underwent imaging with multidetector row computed tomography (MDCT) and cancer staging. To obtain histological confirmation of pancreatic carcinoma, brushing cytology of the pancreatic duct stricture and pancreatic juice cytology were performed at the time of biliary drainage. When histological confirmation was not possible via endoscopic retrograde cholangiopancreatography, we performed endoscopic ultrasound fine-needle aspiration (EUS-FNA). Histological confirmation was obtained for all subjects prior to NAC.

**Figure 1:**
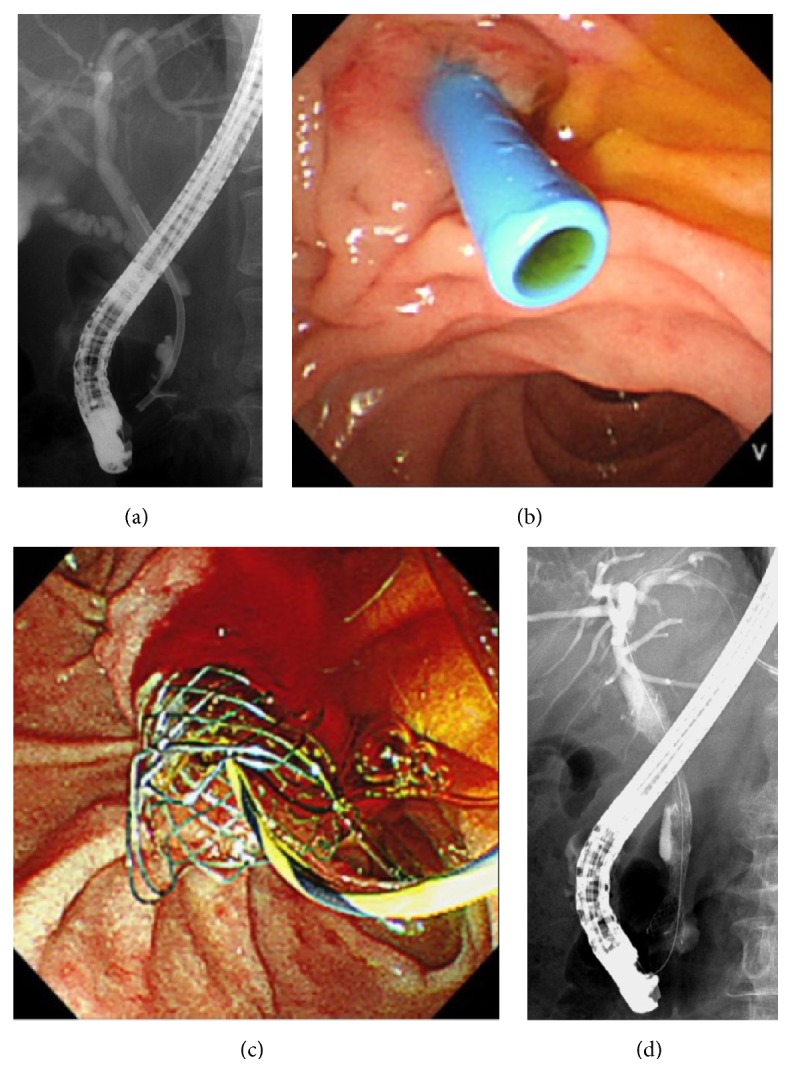
Endoscopic biliary drainage. (a), (b) X-ray image and endoscopic image of plastic stent. (c), (d) X-ray image and endoscopic image of metallic stent.

As a rule, endoscopic nasobiliary drainage was the first drainage procedure performed before histological confirmation was obtained. After histological confirmation was obtained, either PS (the end of May 2013) or MS (as of June 2013) was inserted. In the PS group, 7 Fr, 7 cm straight type biliary drainage stent (Flexima, Boston Scientific) was placed. In the MS group, 10 mm, 6 cm fully covered metallic stent (WallFlex, Boston Scientific) was placed (Figure 1). Two subjects had transferred from other hospitals with a PS already inserted and retained the PS after EUS-FNA.

### 2.3. Definition of BR and Locally Advanced Unresectable Pancreatic Cancer

**Figure 2:**
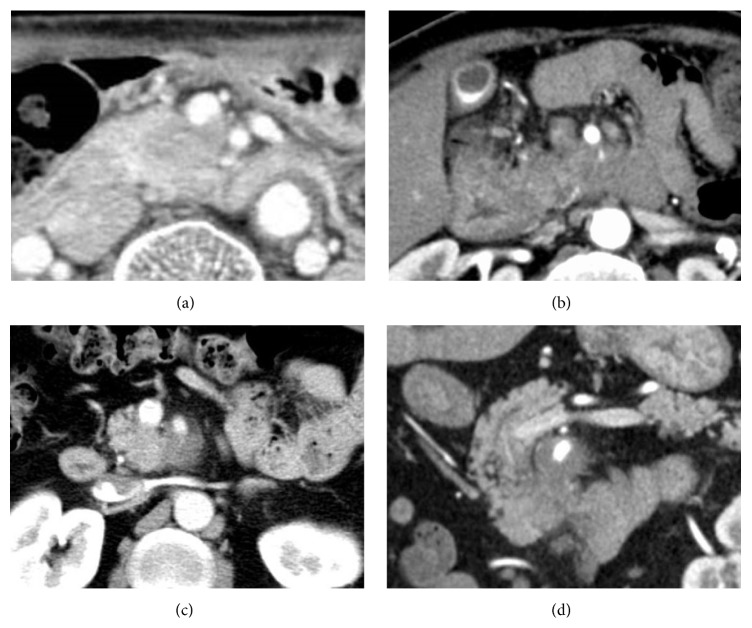
Representative computed tomography images of borderline resectable pancreatic cancer and locally advanced unresectable pancreatic cancer. (a), (b) Tumor in the pancreatic head slightly abutting the portal vein and invading less than half of the circumference of the superior mesenteric artery (borderline resectable pancreatic cancer). (c), (d) Tumor in the pancreatic head invading less than half the circumference of the superior mesenteric artery (locally advanced unresectable pancreatic cancer).

According to the NCCN guideline, BR tumors are defined by tumor abutment (≤180° or ≤50% of the vessel circumference) of the superior mesenteric artery (SMA) or celiac axis, short segment abutment or encasement (>180° or >50% of the vessel circumference) of the common hepatic artery (typically at the gastroduodenal artery origin) that is amenable to segmental resection and primary repair, or segmental venous occlusion with an adequate superior mesenteric vein (SMV) below and portal vein (PV) above the area of tumor-induced occlusion to allow for interpositional grafting [19, 20] (Figure 2).

### 2.4. NAC Protocol and Response Evaluation

We administered three cycles of gemcitabine and S-1 combination therapy (GS therapy) for BR pancreatic cancer. Gemcitabine 1000 mg/m^2^ was administered on days 1 and 8, while S-1 80 mg/body (<1.25 m^2^/body surface area) or 100 mg/body (≥1.25 m^2^/body surface area) was administered on days 1–14. After the three cycles were completed, MDCT was performed. Surgery was performed on cases that were classified as showing stable disease (SD) or a better response according to the Response Evaluation Criteria in Solid Tumors (RECIST).

In all cases, there was a 3-week waiting period between the completion of NAC and surgery.

### 2.5. Basis for Determination of the Number of Cases and Statistical Analysis

Based on previous reports on PBD with NAC, we assumed that the onset of complications caused by bile drainage would be 65% for PS and 10% for MS. Therefore, we set the alpha error at 0.05 and detectability at 80%, which resulted in nine required subjects per group (for a total of 18).

Statistical analysis was performed with the Mann-Whitney *U* test and the chi square test. Statistical significance was set at *p* < 0.05.

## 3. Results

### 3.1. Patients' Clinical Characteristics

PBD was performed on 11 subjects in the PS group and nine subjects in the MS group. The overall median age was 64.5 years. The male-to-female ratio was 12 : 8. All patients underwent completion pancreaticoduodenectomy. Six subjects underwent R0 surgery in the MS group, as did six in the PS group (*p* = 0.66). There were no significant differences between groups in any other patient background factor (Table 1).

### 3.2. Delayed NAC and Rate of Complications Caused by PBD

Our investigation of delayed NAC due to all types of complications indicated that nine subjects in the PS group and one subject in the MS group experienced delayed NAC (*p* = 0.01). Of these, one subject in the PS group and one subject in the MS group experienced delayed NAC due to bone marrow suppression. Significantly fewer patients in the MS group versus the PS group experienced delayed NAC due to PBD (0 versus 8, resp.; *p* = 0.01).

The rate of complications caused by PBD was 0% in the MS group (0/9) and 72.7% in the PS group (8/11). Specifically, occlusion occurred five times, migration occurred two times, and retrograde cholangitis occurred one time (*p* = 0.01; Table 2).

**Figure 3:**
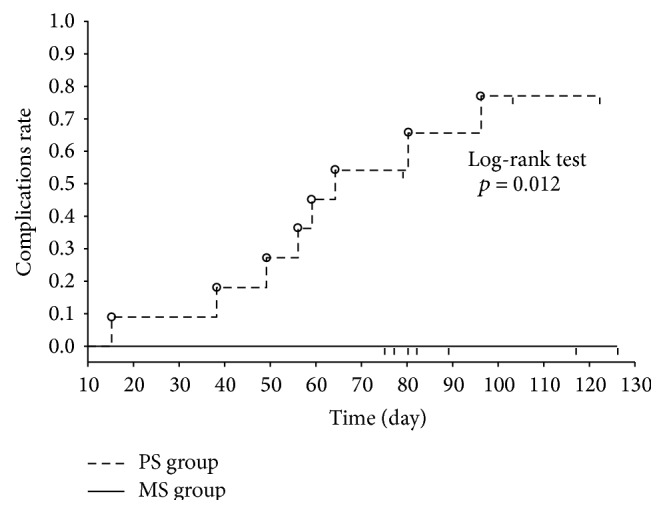
Cumulative rate of complications by stent type. Cumulative rate of complications was investigated using the Kaplan-Meier method. The rate of complications on day 60 was 45% for PS and 0% for MS. On day 90, it was 60% for PS and 0% for MS. Cumulative rate for complications was significantly lower for MS than for PS. Statistical analysis was performed using the log-rank test. MS, metallic stent; PS, plastic stent. The solid line is the MS group; the wavy line is the PS group.

Our investigation of the cumulative rate of complications caused by PBD determined with the Kaplan-Meier method indicated that the rate was 0% on days 60 and 90 in the MS group and 45% on day 60 and 60% on day 90 in the PS group, indicating a significantly lower rate in the MS group (log-rank test, *p* = 0.012; Figure 3).

### 3.3. Effect of Drainage Stents on Perioperative Factors

Blood loss in the MS and PS groups was 960 mL and 1450 mL (*p* = 0.24), respectively; the operative time was 364 minutes and 469 minutes (*p* = 0.24), respectively; and the length of postoperative hospitalization was 18 days and 21 days (*p* = 0.13), respectively. There were two cases of postoperative complications in the PS group (18%): specifically, one case of hepatic necrosis and one case of cholangitis. There were three cases of postoperatibe complications in the MS group (29%): specifically, one case of intra-abdominal abscess, one case of cholangitis, and one case of intestinal necrosis (*p* = 0.61), indicating that there was no significant difference in perioperative factors (Table 3).

### 3.4. Cost of Hospitalization from Diagnosis until Surgery

We investigated seven subjects from each group, excluding two subjects in the PS group whose costs could not be calculated because they were transferred from other hospitals. The median cost of hospitalization (*USD*) was 1,3650 in the PS group and* USD* 1,0580 in the MS group, indicating no significant difference between the groups (*p* = 0.19; Table 3).

## 4. Discussion

Since MS used in PBD may have a negative effect (adhesion to the bile duct and vessels) on surgery and since the per-stent cost is high, PS are generally used in PBD procedures. Recent studies have reported on cases in which neoadjuvant therapy was administered to patients with BR to improve the long-term outcome of pancreatic cancer. If the period until surgery is delayed as a result of neoadjuvant therapy, there is an increased frequency of procedural accidents related to bile duct drainage, such as stent occlusion and cholangitis, which leads to the clinical problem of an increase in the number of cases in which surgery and neoadjuvant therapy must be delayed [21]. Mullen et al. reported that stent-related procedural accidents during NAC occurred in 45% of PS cases (75/166) and 7% of MS cases (2/29), while Wasan et al. reported the same in 93% of PS cases (39/42) and 15.3% of MS cases (2/13) [22, 23]. Aadam et al. reported that the rate of complications caused by drainage during NAC when MS were used for bile duct drainage was 15% on day 260 and that stable drainage was possible [24]. Our study findings also indicated that complications considered stent-related procedural accidents did not occur when MS were used but occurred in 72.7% of cases in which PS were used. Comparison of the results of the present and previous studies suggests that there are fewer drainage-related procedural accidents during NAC when MS are used for PBD.

The effect of MS use in the perioperative phase of PBD procedures has not been elucidated and few studies have reported on its effect in surgery [22, 25, 26]. In their investigation of different stent types used in preoperative drainage procedures, Mullen et al. reported no difference between PS and MS use in operative time, blood loss, hospitalization period, postoperative mortality, or perioperative complications [22]. Our study also found no significant differences in terms of operative time, blood loss, or postoperative hospitalization. We believe that these results suggest that the use of MS in PBD procedures has little influence in the perioperative phase.

There have been almost no studies of the one drawback associated with the use of MS in PBD procedures: its cost. In their investigation of the cost from the start of PBD until surgery, Kubota et al. reported that the use of PS in PBD procedures cost USD 11,545 and that the use of MS cost USD 11,773, indicating no significant difference between the two [27]. A simulation of individual cases revealed that PS placement with two or more reinterventions was more effective than a single MS placement [27]. Our study also indicated no significant cost difference associated with the use of different stent types in PBD procedures. These results suggest that, compared to the use of PS, the use of MS in PBD procedures is not disadvantageous in terms of medical cost.

Finally, we obtained extremely interesting results from our investigation of the R0 resection rate. When NAC was performed at our facility, the R0 resection rate was 44.4% when PS was used and 85.7% when MS was used. Several studies reported that the R0 resection rate improves after neoadjuvant therapy [13, 28, 29]. McClaine et al. [26] reported that performing NAC on BR pancreatic cancer increased the R0 resection rate to 67%, and Kubota et al. [27] reported that neoadjuvant chemoradiation therapy for the treatment of BR pancreatic cancer increased the R0 resection rate to 97% (33/34). However, there have been no studies on the effect that different stent types used in PBD have on the R0 resection rate. We believe the following to be the likely reason for the increase in the R0 resection rate due to the use of MS. Because MS use is associated with fewer complications caused by bile duct drainage, it is likely that a sufficient amount of anticancer agent can be intensively administered in a short amount of time to maximize tumor shrinkage. However, since this study involved only a small number of subjects, this issue will require further studies with larger numbers of subjects.

Because this study was limited by its retrospective and nonrandomized design in a single facility, future multi-institution and prospective randomized controlled trials will be necessary.

In conclusion, this study elucidated the fact that since the use of MS in PBD procedures does not lead to increased perioperative procedural accidents and significantly decreases the onset of complications that occur in association with bile duct drainage during chemotherapy, it can contribute to safer treatment. Although its per-unit cost is high, its reintervention rate is low. This suggests that, compared to PS, MS use may not be disadvantageous in terms of medical cost. This suggests the superiority of MS as the first choice in PBD performed in conjunction with NAC.

## 5. Conclusion

Metallic stent implantation may be effective for preoperative biliary drainage for pancreatic cancer treated with NAC.

## Figures and Tables

**Table 1:** Patients' baseline clinical characteristics.

	MS group	PS group	*p* value
Patients, *n*	9	11	
Median age, years	63	65	0.58^*∗*^
Sex, M/F	7/2	5/6	0.19^*∗∗*^
Tumor size, mm	25.5	27	0.5^*∗*^
Portal vein resection, ±	7/2	7/4	0.64^*∗*^
Artery resection, ±	2/7	3/8	1^*∗∗*^
R0/R1	6/3	6/5	0.66^*∗*^

MS, metallic stent; PS, plastic stent. Statistical analysis was performed using the Mann-Whitney *U* test *∗* and the 2 × 2 chi square test *∗∗*.

**(a):** 

	All complications		
	MS group	PS group	*p* value
Delayed NAC	1	9	
Nondelayed NAC	8	2	0.01

**(b):** 

	Biliary stent complication		
	MS group	PS group	*p* value
Delayed NAC	0	8	
Nondelayed NAC	9	3	0.01

MS, metallic stent; PS, plastic stent

Statistical analysis was performed using the 2 × 2 chi square test.

**Table 3:** 

	MS group	PS group	*p* value
Blood loss (mL, range)	960 (303–3593)	1450 (547–2830)	0.24
Operation time (min, range)	364 (300–686)	469 (308–552)	0.24
Postoperatibe hospital stay (day, range)	18 (17–29)	21 (17–33)	0.13
Cost (US dollar, range)	10580 (7907–28977)	13650 (8444–54534)	0.19

MS, metallic stent; PS, plastic stent. Statistical analysis was performed using the Mann-Whitney *U* test.
